# The Influence of Catalysis on Mad2 Activation Dynamics

**DOI:** 10.1371/journal.pbio.1000010

**Published:** 2009-01-13

**Authors:** Marco Simonetta, Romilde Manzoni, Roberto Mosca, Marina Mapelli, Lucia Massimiliano, Martin Vink, Bela Novak, Andrea Musacchio, Andrea Ciliberto

**Affiliations:** 1 Department of Experimental Oncology, European Institute of Oncology, Milan, Italy; 2 IFOM, Istituto FIRC di Oncologia Molecolare, Milan, Italy; 3 Oxford Centre for Integrative Systems Biology, University of Oxford, Oxford, United Kingdom; 4 Research Unit of the Italian Institute of Technology (IIT) Foundation at the IFOM-IEO Campus, Milan, Italy; Dana-Farber Cancer Institute, United States of America

## Abstract

Mad2 is a key component of the spindle assembly checkpoint, a safety device ensuring faithful sister chromatid separation in mitosis. The target of Mad2 is Cdc20, an activator of the anaphase-promoting complex/cyclosome (APC/C). Mad2 binding to Cdc20 is a complex reaction that entails the conformational conversion of Mad2 from an open (O-Mad2) to a closed (C-Mad2) conformer. Previously, it has been hypothesized that the conversion of O-Mad2 is accelerated by its conformational dimerization with C-Mad2. This hypothesis, known as the Mad2-template hypothesis, is based on the unproven assumption that the natural conversion of O-Mad2 required to bind Cdc20 is slow. Here, we provide evidence for this fundamental assumption and demonstrate that conformational dimerization of Mad2 accelerates the rate of Mad2 binding to Cdc20. On the basis of our measurements, we developed a set of rate equations that deliver excellent predictions of experimental binding curves under a variety of different conditions. Our results strongly suggest that the interaction of Mad2 with Cdc20 is rate limiting for activation of the spindle checkpoint. Conformational dimerization of Mad2 is essential to accelerate Cdc20 binding, but it does not modify the equilibrium of the Mad2:Cdc20 interaction, i.e., it is purely catalytic. These results surpass previously formulated objections to the Mad2-template model and predict that the release of Mad2 from Cdc20 is an energy-driven process.

## Author Summary

Mitosis, the partition of chromosomes from a mother cell to the two daughter cells, is based on the formation of attachments between chromosomes and the mitotic spindle. Cells enter mitosis with replicated chromosomes (sister chromatids) that are held together by a cohesive force. Upon attachment of the sister chromatids to the mitotic spindle, the cohesive force that holds them is removed, and the sisters are parted to opposite poles of the spindle. It is essential for the long-term viability of cells that chromosomes not be lost in the process. For this purpose, cells have evolved a molecular device (the spindle assembly checkpoint or SAC), which prevents loss of sister chromatid cohesion until all sister chromatids are properly attached to the mitotic spindle. An outstanding question concerns the way the SAC signal is amplified away from chromosomes that are not yet attached to the spindle. Such an amplification mechanism has been predicted on the fact that as few as a single unattached kinetochore is able to prevent sister chromatid cohesion. In this paper, we show that the properties of the SAC protein Mad2 are ideally suited to provide a mechanism of amplification to the SAC.

## Abstract 3

The reconstitution in vitro of key reactions of the spindle assembly checkpoint reveals the presence of catalysis and autocatalysis during checkpoint activation.

## Introduction

The process of mitosis is designed to deliver the faithful, equational division of the replicated genome. The generation of tightly connected replicated chromosomes (sister chromatids) during S-phase is prerequisite to the division process. Sister chromatid cohesion is then removed synchronously and irreversibly at the so-called metaphase-to-anaphase transition, when the individual sisters are distributed to the daughter cells in two equal masses [1].

Before sister chromatid cohesion is removed, every pair of sister chromatids must have achieved bipolar orientation at the metaphase plate of the mitotic spindle. A biochemical device named the spindle assembly checkpoint (SAC) exercises tight control over the timing of anaphase, and ensures that anaphase only takes place after all sister chromatid pairs are bioriented [2]. The loss of sister chromatid cohesion at the metaphase-to-anaphase transition requires the activity of the Ub-ligase anaphase-promoting complex/cyclosome (APC/C) and of its activator Cdc20 [3]. To synchronize anaphase with the completion of bipolar orientation of all sister chromatid pairs, the SAC targets and inactivates Cdc20. Two SAC components, the Mad2 protein and the BubR1:Bub3 complex (we indicate a complex of two or more proteins with the names of the proteins separated by a colon), are able to bind Cdc20 directly [2]. Together, these proteins form the mitotic checkpoint complex (MCC), which is thought to act as a pseudosubstrate inhibitor of the APC [4,5].

Central questions in the checkpoint field concern the mechanism of checkpoint activation early in mitosis and the mechanism of checkpoint inactivation prior to anaphase. Unattached kinetochores are believed to play a major role in SAC activation [2]. Kinetochores are complex protein scaffolds assembled on mitotic chromosomes and are responsible for microtubule capture [6]. Kinetochores of sister chromatid pairs that have not attained bipolar attachment are responsible for checkpoint activation and maintenance [7]. Consistently, all key players of the SAC (including, among others, Mad1, Mad2, Mps1, Bub1, BubR1, Bub3, and Cdc20 itself) localize at unattached prometaphase kinetochores [2]. Eventually, once the last kinetochore has attached, Cdc20 becomes able to activate the APC towards cyclin B and securin, its critical substrates at the metaphase to anaphase transition, and the cell exits from mitosis [3].

**Figure 1:**
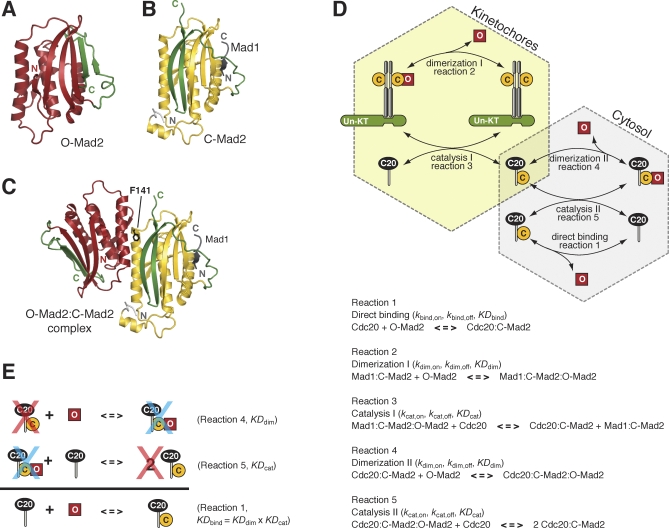
Chemical Reactions That Control the Activation of the SAC (A) Ribbon model of O-Mad2 [39]. Ribbon models were obtained with PyMol, by DeLano Scientific (http://www.pymol.org). The invariant core of the structure (N) is shown in red. The C-terminal mobile element (C), known as the “safety belt” [22], is in green. (B) Ribbon model of C-Mad2. The core of the structure is coloured yellow, with the C-terminal tail and safety belt in green. A segment of Mad1 that stabilizes the C-Mad2 conformation is shown in grey [22]. (C) Ribbon diagram of the O-Mad2:C-Mad2 asymmetric dimer with same colour codes as (A) and (B) [14]. (D) The Mad2 template model [13]. O-Mad2 and C-Mad2 are represented with red squares and yellow circles, respectively. Mad1 is represented with grey cylinders. The Mad2 binding site in Mad1 and Cdc20 is shown as a thin grey cylinder. The light-yellow hexagon includes all the reactions taking place at unattached kinetochores (Un-KT), while the grey hexagon includes cytosolic reactions. Cdc20:C-Mad2 is the only chemical species that belongs to both sets. The reactions describe binding (1), dimerization (2 and 4), and catalysis (3 and 5). An underlying hypothesis of these reactions is the presence of a highly unstable form of active Mad2, I-Mad2, more prone to bind Cdc20 than O-Mad2. For the sake of simplicity, we do not include it explicitly in our reaction scheme. In Table S1, we report the differential equations formalizing the reaction network. (E) The reactions of dimerization and catalysis form a closed loop that produces the binding reaction. Since no energy is introduced into the system, microscopic reversibility applies, and the hypothetical reaction does not affect the equilibrium of the system.

The mechanism whereby unattached kinetochores regulate the binding of Mad2 to Cdc20, possibly the first step in the assembly of the MCC, is still unclear. Mad2 comes in two different conformations, open (O-Mad2) and closed (C-Mad2), that differ for the position of the C-terminal tail (Figure 1A and 1B, reviewed in [8]). Before SAC activation, most Mad2 is found in the monomeric open form and is not bound to Cdc20. Upon SAC activation, O-Mad2 binds to its Cdc20 target and switches to the closed form [9,10].

Throughout the cell cycle, a remaining 10%–25% of Mad2 is engaged in a very stable complex with Mad1 [10–12] in which Mad2 holds the C-Mad2 conformation (Figure 1B). The role of the Mad1:C-Mad2 complex in the sequestration of Cdc20 by Mad2 is underlined by four key observations. First, the tight Mad1:C-Mad2 complex acts as the kinetochore receptor of cytosolic O-Mad2, via the “conformational” dimerization of C-Mad2 and O-Mad2, a reaction that creates the trimer Mad1:C-Mad2:O-Mad2 (Figure 1C) [13,14]. Second, Mad1 is required for Mad2 to bind Cdc20, at least in a normal cell cycle (reviewed in [2]). Third, Mad2 mutants that are able to bind Mad1, but that are impaired in the formation of Mad2 conformational dimers, cannot complement the SAC defect when expressed in a *mad2-*deleted strain [13,15]. Fourth, Mad1:C-Mad2 has been recently shown to convert O-Mad2 into C-Mad2, and evidence for its role in the formation of Cdc20:C-Mad2 has been provided [16].

On the basis of these data, it has been suggested that Mad1-bound C-Mad2 starts a catalytic amplification of the checkpoint by binding to O-Mad2 in the Mad1:C-Mad2:O-Mad2 trimer, and thus facilitating the conversion of O-Mad2 into Cdc20-bound C-Mad2 (the network is described by reactions 1–3 in Figure 1D). This still speculative model of Mad2 activation is named the Mad2-template model [13]. A possible twist to the model comes from the observation that Cdc20-bound C-Mad2 is a structural copy of Mad1-bound C-Mad2. Once released in the cytosol, Cdc20-bound C-Mad2 (located on the overlap between the yellow and the grey hexagons in Figure 1D) might therefore propagate the O-Mad2 conversion away from kinetochores through an autocatalytic loop (Figure 1D, reactions 4 and 5).

As the molecular mechanisms of the SAC are investigated at increasingly deeper detail, it becomes progressively more attractive to develop mathematical models to rationalize SAC behaviour and to predict the effects of its manipulation. Recent theoretical studies proposed that the autocatalytic loop of the Mad2-template model would force the cell to be permanently arrested in a state of operational SAC, with most Cdc20 sequestered by Mad2 [17,18]. Other studies argued that the contribution of the autocatalytic loop to the SAC is negligible [19]. The previous studies, however, have neglected two fundamental kinetic and thermodynamic implications of the conversion of Mad2 from the open to the closed conformation. The kinetic implication is that the dramatic structural rearrangement of Mad2 can be expected to translate in a very slow, natural on-rate of binding to Cdc20. Were this true, the acceleration predicted by the “template” might be required to accelerate the formation of Mad2:Cdc20 complexes required to halt progression into anaphase. The thermodynamic implication is that the template model, as it is cast in Figure 1D, does not imply irreversible reactions (Figure 1E). Thus, the rate equations of the template model describe the influence of Mad2 dimerization on the rate at which the Mad2:Cdc20 complex forms, but do not imply a modification of the equilibrium concentrations of the Mad2:Cdc20 complex.

Here, we show through a combination of experimentation and mathematical modelling that catalytic amplification of Mad2:Cdc20 complex formation is required as a first step of checkpoint activation to overcome the kinetic barrier built in the Mad2:Cdc20 interaction.

## Results

### The Basal Rate of Binding of Cdc20 and Mad2 Is Slow

To gain insight into the basal rate of the interaction of O-Mad2 and Cdc20 (reaction 1 in Figure 1D), it is important to remove the possible effects of dimerization. Previously, we have described several Mad2 point mutants that are impaired in dimerization, but that retain the ability to bind to Cdc20 in vitro [13,15,20,21]. In one such mutant, Phe141 of Mad2 is replaced by Ala (Mad2^F141A^). Mad2^F141A^ is unable to sustain the “conformational” dimerization of Mad2 and is unable to complement a *mad2* deletion strain of Saccharomyces cerevisiae [15,21].

**Figure 2:**
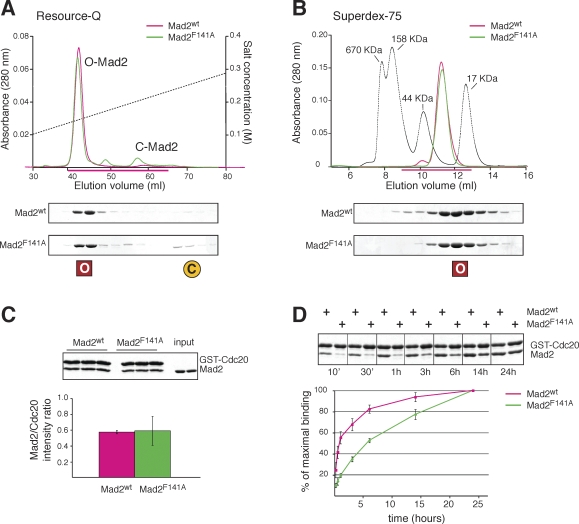
Mad2^wt^ Binds Cdc20 Faster Than Mad2^F141A^ (A) Chromatographic analysis of Mad2^wt^ and Mad2^F141A^. In agreement with previous studies [10,14], both Mad2 species can be purified in an O-Mad2 conformation that is identified based on the salt concentration (dotted line) at which these species elute from an anion exchange column. Mad2 species in the C-Mad2 conformation (yellow circle) elute at higher salt concentrations relative to the O-Mad2 species (red square) [10,14]. (B) Mad2^wt^ and Mad2^F141A^ were separated on a Superdex-75 10/30 column. Both proteins elute as expected for monomeric forms. Dotted line represents the elution volumes of gel filtration standards. (C) GST-Cdc20^111−138^ (at 1 μM total concentration) was immobilized on GSH-agarose beads, and incubated for 24 h at room temperature with 1 μM Mad2^wt^ or Mad2^F141A^. The binding reactions were then analysed by SDS-PAGE. Band intensities were quantified by densitometric analysis, and the ratio between GST-Cdc20 and Mad2 bands were used to calculate the fraction of Mad2/Cdc20 complexes. Standard deviations (error bars) were calculated from experiments repeated three or more times. (D) The experiment described in (C) was carried out as a time course using 1 μM GST-Cdc20^111−138^ and 2 μM Mad2^wt^ or Mad2^F141A^. SDS-PAGE gels were digitized, and the intensity of the bound fractions plotted as a function of time. As in (B), error bars indicate the standard deviation calculated from three or more experiments.

In a first set of experiments, we sought evidence that wild-type Mad2 (Mad2^wt^) and Mad2^F141A^ bind Cdc20 with similar affinity. This expectation is sensible when considering that Phe141 is localized at the interface between O-Mad2 and C-Mad2, and that the dimerization interface occupies the opposite end of Mad2 from where Cdc20 binds (Figure 1A and 1B). By using recently described approaches, Mad2^wt^ and Mad2^F141A^ were purified to homogeneity in a monomeric state and in the O-Mad2 conformation (Figure 2A and 2B). A 1 μM concentration of these species was then incubated with a 1 μM concentration of a construct encompassing the Mad2-binding region of Cdc20 fused to glutathione S-transferase (GST; Figure 2C). Indeed, at equilibrium (i.e., after a 24-h incubation), we found similar amounts of Mad2^wt^ and Mad2^F141A^ on the GST-Cdc20 beads, indicating that Mad2^F141A^, like other Mad2 mutants that are impaired in conformational dimerization, has a very similar binding affinity for Cdc20 as Mad2^wt^ (Figures 2C and S1).

We therefore proceeded to analyze the rate of binding of Mad2^wt^ and Mad2^F141A^ to GST-Cdc20. Mad2^wt^ had reached maximal binding between 1 and 3 h. Conversely, it took Mad2^F141A^ between 12 and 24 h to reach maximal binding (Figure 2D). These results suggest that the abrogation of the ability of Mad2 to form conformational dimers slows down the binding to Cdc20 in this assay. Indistinguishable results were obtained with another Mad2 dimerization mutant, Mad2^R133A^ (unpublished data).

In summary, the fact that Mad2^F141A^ is impaired in Mad2 conformational dimerization, and that its overall binding affinity to Cdc20 is unchanged relative to Mad2^wt^, supports our argument that the rate of binding of Mad2^F141A^ to Cdc20 represents the basal rate of binding of Mad2 to Cdc20 in the absence of Mad2 dimerization. Strong additional evidence in favour of this proposition is provided in the next sections.

### A New Assay to Measure Binding Kinetics

**Figure 3:**
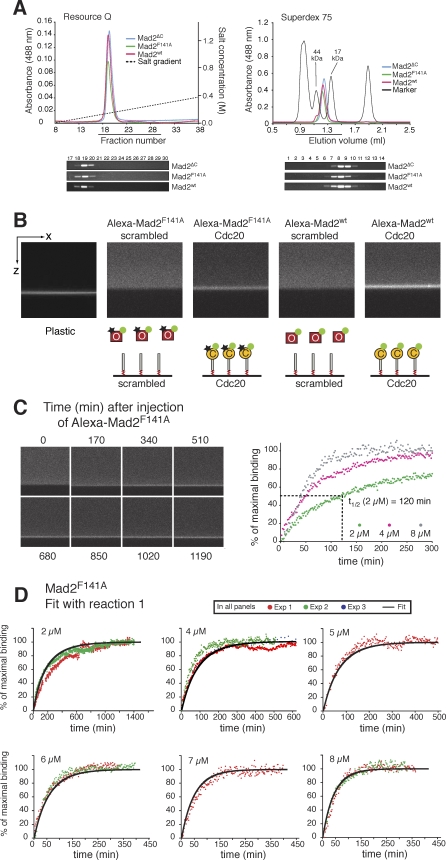
Kinetic Analysis for Rate Constants Determination of Mad2^F141A^ (A) The different Mad2 species used in the analysis retained their O-Mad2 conformation (left) and monomeric state (right) after covalent labelling with Alexa Fluor 488. After SDS-PAGE separation, the Alexa-labelled species were visualized under a UV transilluminator. (B) A flow chamber was built in which a biotinylated Cdc20 peptide (∼1 μM Cdc20, measured as the moles of peptide bound onto the surface divided by the volume of the chamber in litres; Figure S4) is immobilized onto the bottom surface through a biotin-streptavidin interaction. After addition of fluorescent Mad2, bound Mad2 can be visualized. The montage shows the specificity of the binding reaction. A black star characterizes Mad1^F141^ as opposed to Mad2^wt^; red squares indicate O-Mad2; yellow circles indicate C-Mad2; and a green dot represents a fluorescent label. (C) Real-time binding experiment using Alexa-Mad2^F141A^. The experiment was carried out at several Mad2 concentrations as indicated in the plot. (D) Fitting of the binding experiment with reaction 1 of Table S1. Parameters that gave the best fitting are reported in Table I. The fitting was carried out contemporarily on all available curves and at different concentrations as described in Text S1. As for the goodness of the fit, see Text S1 and Figure S5.

To quantify the association rate between Cdc20 and Mad2 in vitro, we developed a real-time assay based on the binding of Alexa Fluor 488–labelled Mad2 (Alexa-Mad2) to a surface containing immobilized Cdc20 in a flow cell. The method is conceptually similar to the Biacore method, but is in principle amenable to multicolour analysis. Alexa-Mad2^F141A^ and Alexa-Mad2^wt^ retained their monomeric O-Mad2 conformation after covalent fluorescent labelling (Figure 3A). As they bind to Cdc20 on the surface of the flow cell, they convert into C-Mad2 (Figure 3B and 3C). As the reaction proceeds, the signal in solution decreases, while the signal on the surface increases. By measuring the fluorescence on the surface (or the signal in solution; Figure S2 and Text S1) by confocal microscopy, we followed the binding kinetics at different concentrations of Mad2^F141A^ (Figure 3C).

We first attempted to interpret the kinetics of Mad2^F141A^ binding to Cdc20 on the basis of reaction 1 in Figure 1D. Following the increase of signal on the surface, we could fit the experimental data over a 4-fold change of [Mad2^F141A^] using *k*_bind,on_ = 4.83 × 10^−5^ μM^−1^ s^−1^ and *k*_bind,off_ = 4.83 × 10^−6^ s^−1^, confirming that the rate constants for the interaction of Mad2^F141A^ are exceptionally small (Figure 3D). By comparison, this on rate is almost four orders of magnitude slower than that of Mad2 dimerization (see below). The small rate constants are consistent with the semiquantitative binding experiments carried out with unlabelled Mad2^F141A^ (Figure 2D). As the dissociation constant (*KD*_bind_) is given by the ratio *k*_bind,off_/*k*_bind,on_, our kinetic analysis predicts a *KD* of 100 nM for the interaction of Mad2^F141A^ with Cdc20, in excellent agreement with previous analyses [9,13,16,22].

### The Formation of Dimers C-Mad2:O-Mad2 Accelerates the Reaction

In summary, the kinetic analysis on Mad2^F141A^ confirms the hypothesis that the Mad2 conformational change is very slow and likely rate limiting for checkpoint activation. Several factors might contribute to accelerate the basal rate of binding revealed by our experiments. For instance, it has been reported that several kinases, including checkpoint kinases, target Cdc20, thus possibly contributing through phosphorylation to its susceptibility to be inhibited by the SAC [23–27].

**Figure 4:**
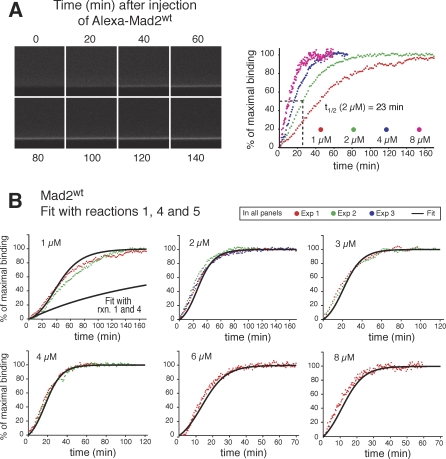
Kinetic Analysis for Rate Constants Determination of Mad2^wt^ (A) Different concentrations of Mad2^wt^ were introduced in a flow chamber containing approximately 1 μM Cdc20. (B) Fitting of the binding curves of Mad2^wt^ with reactions 1, 4, and 5 (Table S1 and Table S2) and parameters in Table I. The fitting was carried out contemporarily on all available curves and at different concentrations as described in Text S1. As for the goodness of the fit, see Text S1 and Figure S5. In the first panel (1 μM), a fit with only reactions 1 and 4 is also shown to demonstrate the importance of adding catalysis (reactions 5) in the fitting process. Notice how for low concentrations of Mad2, the kinetics follows a sigmoidal increase reflecting the slow initial binding of Mad2^wt^ to Cdc20.

Although there might be numerous additional factors impinging on the velocity of formation of Mad2:Cdc20 complexes, we decided to test the concept that conformational dimerization of Mad2 is important to accelerate this binding reaction, as advocated by the Mad2 template model. To measure the effect of dimerization in the binding between Mad2 and Cdc20, we repeated the real-time binding experiments with Mad2^wt^, which—compared to Mad2^F141A^—has the ability to dimerize. In agreement with our hypothesis, the half-time of binding of Mad2^wt^ to Cdc20 at 2 μM Mad2 was five to six times faster than that of Mad2^F141A^ (Figure 4A). The binding kinetics were sigmoidal, typical for an autocatalytic reaction with a slow initial phase dominated by the binding of O-Mad2 to Cdc20, which is followed by a faster reaction based on activation by Mad2 dimerization.

**Figure 5:**
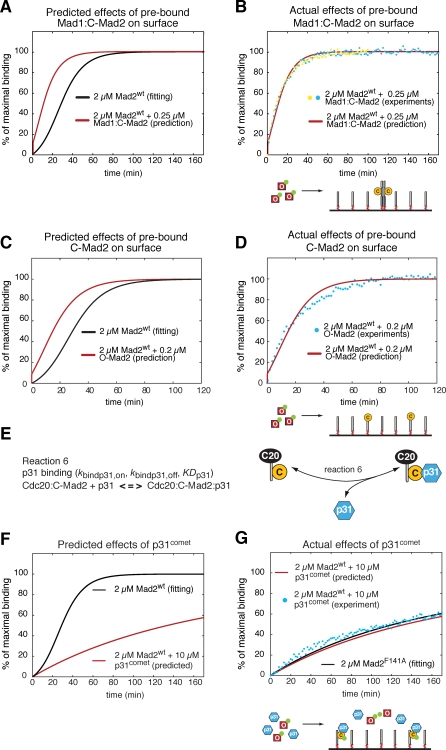
Testing the Predictions of the Model (A) The time course of accumulation of Mad2^wt^ (at 2 μM concentration) on a surface exposing Cdc20 is shown through the fit to the experimentally determined binding curves (black line). The red line is a prediction of the effects on Mad2^wt^ binding to the surface due to the presence of 0.25 μM Mad1:C-Mad2 interspersed with Cdc20. The prediction is that the reaction is accelerated because Mad2^wt^ can now rapidly bind to C-Mad2 in the Mad1:C-Mad2 complex, as a consequence of which, its transfer to Cdc20 is accelerated. Parameters are listed in Table 1, equations in Tables S1 and S2. (B) Experimental determination (curves defined by yellow and blue dots) of the binding of 2 μM O-Mad2^wt^ to the Cdc20-exposing surface in the presence of Mad1:C-Mad2. There is excellent agreement between the prediction (red curve, the same shown in [A]) and the experiments. Red squares indicate O-Mad2; yellow circles indicate C-Mad2; green dots indicate fluorescent labels. (C) Similarly to (A), the model predicts that the rate of Cdc20:C-Mad2 binding can be accelerated by the presence of preformed Cdc20:C-Mad2. Black solid line: the fit to the experimentally determined binding curves; in red: the predicted timing of Cdc20:C-Mad2 formation in presence of pretreatment of the surface with 0.2 μM Mad2^wt^. Parameters are listed in Table 1, equations in Tables S1 and S2. (D) Experimental results confirm the prediction of the model. The chamber was pretreated with 0.2 μM nonfluorescent Mad2^wt^ until the reaction reached equilibrium. The remaining Mad2^wt^ was washed from the chamber, and 2 μM fluorescent Mad2^wt^ were added in solution. There is excellent agreement between the prediction (red curve, the same shown in [A]) and the experiments (blue dots). (E) The role of p31^comet^ in the SAC is based on its ability to interact with C-Mad2 bound to either Mad1 or Cdc20 in a manner that is competitive with the binding of O-Mad2. p31^comet^ is a negative regulator of the SAC [21,29,37,47]. (F) The black line shows the same time course of accumulation of Mad2^wt^ (at 2 μM concentration) on a surface exposing Cdc20 that was shown in (A). The red line indicates a prediction of the effects on Mad2^wt^ binding to the surface exposing Cdc20 of adding 10 μM p31^comet^. (Parameters are listed in Table 1, equations in Tables S1 and S2.) The prediction is that the reaction is strongly delayed because p31^comet^ binds tightly to the Cdc20:C-Mad2 complex on the surface and prevents the recruitment of additional Mad2^wt^ through dimerization and catalysis. (G) The prediction in (D) is fully satisfied by experimental determination of the binding of 2 μM Mad2^wt^ to Cdc20 in the presence of 10 μM p31^comet^ (blue dots). The time course of Mad2^wt^ is strongly delayed by p31^comet^, and it now resembles the rate observed in the presence of 2 μM Mad2^F141A^, whose fitting is shown with a black line. P31, p31^comet^.

We asked whether we could account for these results in silico by decomposing the reaction into three steps: binding, dimerization, and catalysis (i.e., reactions 1, 4, and 5 in Figure 1D.) Based on the fact that Mad2^F141A^ and Mad2^wt^ have the same affinity for Cdc20 (Figures 2C and S1), *k*_bind,on_ and *k*_bind,off_ were assigned values previously determined for Mad2^F141A^. Association and dissociation constants for dimerization, reaction 4, have been chosen to be compatible with the values measured experimentally: 0.3 μM^−1^ s^−1^ and 0.45 s^−1^, respectively [13,28] (Table 1). Instead, the rates of the hypothetical catalytic reaction (reaction 5) are unknown. However, it should be noted that reactions 4 and 5 form a closed loop whose net result is reaction 1 (Figure 1E). Furthermore, these two reactions give rise to an autocatalytic reaction whereby Cdc20:C-Mad2 induces its own synthesis. Due to the principle of microscopic reversibility, *KD*_cat_ = *k*_cat,off_/*k*_cat,on_ = *KD*_bind_/*KD*_dim_, which implies that the only unknown parameter in our fitting is *k*_cat,on_. We find good fitting to the experimentally determined curves when we set *k*_cat,on_ = 3.0 × 10^−3^ μM^−1^ s^−1^ (Figure 4B and Table 1). This value is approximately two orders of magnitude larger than *k*_bind,on_, the noncatalysed reaction that leads to Cdc20 sequestration. Importantly, if we simply model the reaction with two terms, where Mad2^wt^ can only bind Cdc20 and form dimers (reaction 1 and 4 in Figure 1D), but cannot catalyse the conversion of O-Mad2 (reaction 5), it is impossible to fit the experimental data (see, for example, the simulation for [Mad2] = 1 μM in Figure 4B). This result strongly implies that additional reactions besides binding and dimerization take place with Mad2^wt^ as opposed to Mad2^F141A^. We note that the basal rate equation (reaction 1, Table S1) is based on kinetic parameters derived from experiments carried out with Mad2^F141A^. Thus, our results provide very strong evidence that the rate of Cdc20 binding by Mad2^F141A^ represents the actual basal rate of Mad2^wt^ binding to Cdc20 in the absence of catalysis.

**Table 1:**
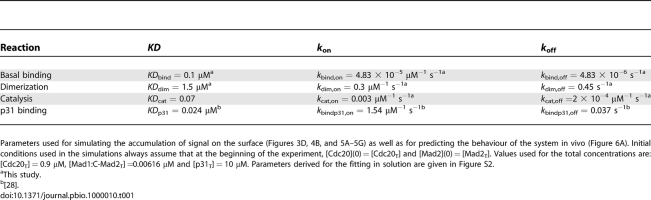
*KD*s, Rate Constants, and Concentrations

**Figure 6:**
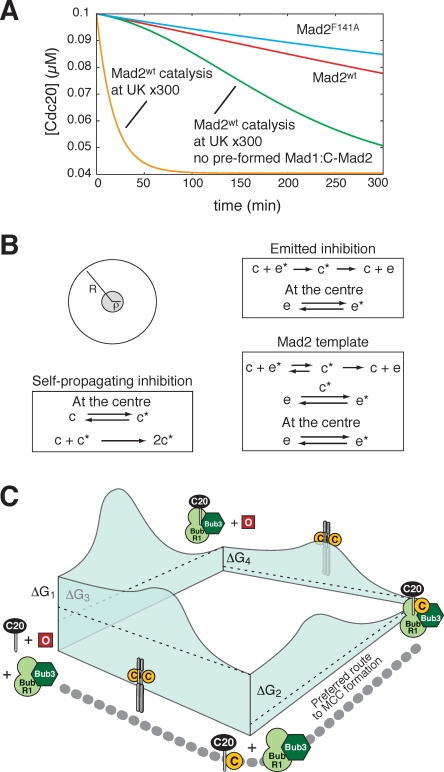
Models of Checkpoint Activation in Living Cells (A) In the presence of physiological concentrations of Mad2, Mad1:C-Mad2, and Cdc20, the full model of Figure 1D (reactions 1 to 5) starts with all Cdc20 and O-Mad2 in the free form. The timing and levels of Cdc20 sequestration in a Cdc20:C-Mad2 complex are plotted. The Mad2^F141A^ mutant (blue line) sequesters Cdc20 extremely slowly. Mad2^wt^ is faster, but still too slow to account for the rapid SAC activation observed in living cells. Only if we arbitrarily assume catalysis at the unattached kinetochores (i.e., at Mad1:C-Mad2) to be 300 times faster in vivo than in vitro (orange line), the activation timing is satisfactory. Only in the presence of preformed Mad1:C-Mad2 can the unattached kinetochores effectively reduce the SAC activation timing. If the Mad1:C-Mad2 complex had to form from Mad1 and O-Mad2, the initial SAC response would be slow even when the catalytic activity of kinetochores (green line) is increased 300 times. Thus, Mad1:C-Mad2 must exist at the beginning of SAC activation for the Cdc20:C-Mad2 complex to accumulate rapidly. Parameters are listed in Table 1, equations in Tables S1 and S2. (B) Graphical representations—adapted from the work of Barkai and collaborators [17]—of different SAC models. The small circle with radius ρ represents a kinetochore in a cell (circle with radius R) in which a given reaction (labelled “at the centre”) takes place. The self-propagating inhibition model and the emitted inhibition model were proposed by Barkai and collaborators [17], whereas the Mad2 template model [13] is described with a formalism similar to that adopted in [17]. Note that here, the only irreversible reaction is the reactivation of the inhibited form of the cell cycle progression protein **c**. The self-propagation model is distinct from the Mad2 template model. (See Discussion for more detailed descriptions of **c**, c*, **e**, and e*.) (C) A speculative representation of the energy profile of the formation of the MCC complex. ΔG_1_ to ΔG_4_ describe Gibbs' free energies for the four reactions described in the diagram. The first step of the preferred route to MCC formation is the formation of the Cdc20:C-Mad2 complex. The reaction is spontaneous, but the activation energy is extremely high. Conformational dimerization catalysed by Mad1:C-Mad2, and unknown additional factors can lower the activation energy, but the equilibrium of the reaction is unchanged. The formation of the MCC complex eventually takes place through the binding of Cdc20:C-Mad2 to a preformed BubR1:Bub3 complex. Red squares indicate O-Mad2; yellow circles indicate C-Mad2.

### Experimental Validation of Model Predictions

Our reaction scheme makes two key predictions. The first prediction is that the binding of O-Mad2 to Cdc20 should occur faster if the C-Mad2 catalyst was present at the beginning of the reaction. This reflects the fact that the natural formation of C-Mad2 is a slow process, and that C-Mad2 accelerates it. To illustrate this point, we compared the fitting curve for the total concentration of Mad2^wt^ equal to two μM (a fitting curve already shown in Figure 4B, which takes into account reactions 1, 4, and 5) with a simulation in which the interaction between Mad2^wt^ and Cdc20 was helped by the presence of Mad1:C-Mad2 via dimerization and catalysis according to reactions 2 and 3 in Figure 1D. (The two curves are shown in black and red in Figure 5A). Due to the structural similarities between Mad1:C-Mad2 and Cdc20:C-Mad2, we assumed that the two species induce catalysis in the same way (i.e., reactions 3 and 5 share the same kinetic parameters), and that the same holds true for dimerization (reactions 2 and 4). The simulations show that the presence of preformed C-Mad2 overrides the lag phase in the binding of Mad2^wt^ to Cdc20, as it provides sufficient initial “catalyst” for the reaction (Figure 5A).

To test the prediction, we monitored the binding of 2 μM Alexa-Mad2^wt^ to approximately 1 μM Cdc20 (a concentration determined by fitting and experimentally, as described in Figure S4 and Text S1) on a surface with interspersed 0.25 μM of Mad1:C-Mad2. Mad1:C-Mad2 forms a very tight complex that cannot undergo significant dissociation within the time frame of our binding experiment [13,20,22,28]. The ratio of free O-Mad2 to Mad1:C-Mad2 chosen for these experiments is similar to that believed to exist in living cells (see [28] and discussion therein), although we suspect that the active concentration of Mad1:C-Mad2, i.e., the pool of this complex that can be recruited to kinetochores, is probably smaller (see below). In quantitative agreement with the simulations, the reaction had no lag phase and produced an overall 3-fold acceleration relative to the noncatalysed rate (Figures 5B and S3). Thus, preformed C-Mad2 accelerates the binding reaction, providing a very strong indication that in the absence of Mad1:C-Mad2, the lag phase represents the slow accumulation of C-Mad2 bound to Cdc20. Similar effects were observed when an initial “seed” of C-Mad2 was created by letting substoichiometric amounts of unlabeled O-Mad2 bind Cdc20 on the surface prior to the addition of labelled O-Mad2 (Figure 5C and 5D).

The second prediction is that any interference with conformational dimerization should reduce the rate of binding of Mad2^wt^ to Cdc20. To test the prediction, we sought to impair the dimerization of Mad2. To this aim, we used p31^comet^, a structural mimic of O-Mad2 that binds to C-Mad2 with an approximately 40-fold higher affinity relative to O-Mad2 and in a manner that is competitive with the binding of O-Mad2 [21,28–30]. The association and dissociation rate constants for the interaction of p31^comet^ with Mad2 have been previously determined [28]. Once this reaction is introduced into our reaction scheme (reaction 6, Figure 5E), the simulations show that the interference with conformational dimerization reduces the rate of binding of Mad2^wt^ to Cdc20 to the rate observed with Mad2^F141A^, as it “poisons” the reaction catalyst by efficiently competing with conformational dimerization (Figure 5F). We tested this prediction by mixing 10 μM p31^comet^ with 2 μM Alexa-Mad2^wt^, and by monitoring the binding of Alexa-Mad2^wt^ to Cdc20 in our real-time binding assay. As shown in Figures 5G and S3, the experiments were in quantitative agreement with the prediction of our model.

### Implications of Binding Rates for Spindle Checkpoint Activation

RNA interference (RNAi) experiments on SAC proteins in HeLa cells have demonstrated that the checkpoint must be already fully active approximately 10–12 min after nuclear envelope breakdown (NEB) [31]. In the absence of Mad2, for instance, HeLa cells undergo a precocious anaphase approximately 10–12 minutes after NEB. At present, we do not know precisely at which point, within the approximately 10–12-minute timeframe, the checkpoint response is mounted. We are also ignorant of the mechanisms that allow cells to remain in mitosis for approximately 10–12 min when the SAC is defective, although it has been proposed that this timing might reflect cyclin A degradation, which must be completed before cells can proceed into anaphase [32,33]. For the purpose of our analysis, we assume that it might take between 5 and 10 min to mount a full SAC response after NEB.

We used the full model in Figure 1D, with the parameters estimated in vitro, to simulate the binding between Mad2 and Cdc20 using published cellular concentrations (0.1 μM for Cdc20 and 0.2 μM for Mad2. See [28] and discussion therein). As for the concentration of Mad1:C-Mad2, we assumed that each unattached kinetochore binds approximately a thousand Mad1:C-Mad2 molecules (a similar estimate has been provided for Cdc20 in PtK1 cells [34,35]). Assuming a system with 22 kinetochores and a total volume of 6 pl in ptK1 cells, we introduced a concentration of active Mad1:C-Mad2 (i.e., localized at the unattached kinetochores) of 22 kinetochores × 0.28 nM = 6.16 nM. This value is based on the assumption that at the beginning of prometaphase, all kinetochores are unattached, and all contribute to the activation of the checkpoint.

First, we run a simulation with Mad2^F141A^, which is unable to form conformational dimers and is unable to sustain the SAC [15,21,36]. The simulation shows that at equilibrium, more than 50% of Cdc20 is sequestered, which is possibly compatible with checkpoint maintenance and thus at odds with the observed checkpoint defect. However, the result can be explained looking at the time course of the reaction: sequestration rate would have a *t*_1/2_ of 9.5 h (Figure 6A, blue line), which is clearly incompatible with rapid checkpoint activation.

The substitution of Mad2^F141A^ with Mad2^wt^, i.e., the addition of dimerization and catalysis as measured in vitro, partly alleviates the problem (*t*_1/2_ of 5.3 h, Figure 6A, red line), but does not resolve it. Thus, the checkpoint response would be unreasonably slow if we were to use the parameters measured in vitro. Incidentally, we notice that if the binding of Mad2 and Cdc20 would reach completion in living cells, the SAC would be constitutively operational, impairing cell viability. We hypothesize that in cells, such a fate is avoided by energy-dependent reactions that induce the release of Cdc20 from Mad2, as suggested recently [37,38].

We can envisage two reasons for the disagreement between our in vitro data and the observations in vivo. First, our simulations might underestimate the actual concentration of active Mad1:C-Mad2. If the kinetochores would serve as a platform where Cdc20 and Mad1:C-Mad2 react, then the local concentrations of these molecules would greatly increase. As a rough estimate, the radius of one kinetochore is 0.2 μm, to give a volume of 3.3 × 10^−5^ pl. This provides a local concentration for Mad1:C-Mad2 and Cdc20 on one unattached kinetochore of 46.6 μM. If we introduce this modification, simulations show that free Cdc20 decreases to less than 50% with *t*_1/2_ of 17 min (unpublished data).

The second reason might be that our model, which is exclusively based on measurements in vitro, lacks additional factors required to accelerate the binding in vivo, and therefore underestimates the extent of the catalytic component of checkpoint activation. For instance, the phosphorylation of Cdc20, which is neglected in our modelling, might be an additional factor of acceleration flanking conformational dimerization [23–27].

We tried to quantify the increase in catalytic efficiency of the SAC machinery at the kinetochore, additionally to that provided by Mad2 dimerization, required to obtain reasonable activation kinetics. Simulations with our model show that when the catalytic potency of the kinetochores is increased 300-fold or more (to 0.9 μM^−1^ s^−1^ or faster) then the checkpoint response can be mounted within the limit of 10 min (Figure 6A, orange trace). The identification of the actual molecular mechanism underlying this necessary increase in catalytic efficiency is a subject for further investigation.

## Discussion

We have tested the implications of Mad2 activation kinetics for the SAC. An important conclusion from our studies is that in cells, the noncatalysed binding of Cdc20 and Mad2, albeit spontaneous, is very slow. This discovery is consistent with the dramatic conformational and topological change undergone by Mad2 when binding to Cdc20 [8,9,14,16,21,22,39].

The detailed molecular mechanism underlying the formation of Cdc20:C-Mad2 is a matter of active research. The notion of an active form of Mad2 primed for the binding to Cdc20, intermediate Mad2 (I-Mad2), is now accepted [8,16], but the exact identity of such a form has not been revealed. Yang and collaborators have previously shown that C-Mad2 is a faster Cdc20 ligand relative to O-Mad2 [16], and we have confirmed this result (M. Simoneta, R. Manzoni, M. Mapelli, A. Musacchio, and A. Ciliberto, unpublished data). Based on this observation, Yang and collaborators have proposed that an empty C-Mad2 might be the intermediate required for the formation of Cdc20:C-Mad2.

We believe that the conformational dimerization of Mad2 might be coupled to a catalytic activity that can accelerate the binding to Cdc20. This hypothesis is consistent with the recent structural and functional analysis of the Mad2 conformational dimer, which indicated that the removal of the N-terminal β1-strand of O-Mad2 required for the formation of C-Mad2 might be the rate-limiting step of the Mad2 conversion [14]. A possible mechanism to explain this effect is that C-Mad2, either bound to Mad1 or Cdc20, forms a dimer with O-Mad2 and releases it as I-Mad2 after using part of the binding energy to impart a conformational change onto O-Mad2. I-Mad2 either reverts to O-Mad2 or binds to Cdc20 with much faster binding rate relative to O-Mad2.

We simplified this scheme of O-Mad2 and Cdc20 binding with the assumption that I-Mad2 is short-lived. In our model, a direct interaction between Cdc20 and either Mad1:C-Mad2:O-Mad2 or Cdc20:C-Mad2:O-Mad2 is needed for the catalytic reaction to occur. This scheme, which is the simplest possible way to introduce catalysis, might be too simple. Nevertheless, numerical simulations account very well for the kinetics of Cdc20 binding, both for Mad2^wt^ and Mad2^F141A^, provided that the association constant of the catalytic reaction, the only unknown parameter in our system, exceeds the simple binding of Mad2^F141A^ by two orders of magnitude.

### A Comparison with Published Models

Barkai and coworkers (Doncic et al. [17]) have discussed a “self-propagating inhibition” model as a possible representation of the Mad2-template model (Figure 6B). In the self-propagating inhibition model, a protein **c** required for cell cycle progression, is turned at the kinetochore into an inactive species c* that can diffuse freely in the cytosol to convert more **c** into c* in an autocatalytic and irreversible process. It is difficult to reconcile the molecular mechanism of Mad2 activation with the formalism of the self-propagating inhibition model, and we therefore will not discuss it here in detail.

Certain aspects of the Mad2-template mechanism can be approximated by an alternative model, the “emitted inhibition” model [17] (Figure 6B). The model posits that kinetochores are responsible for the activation of a species **e** to an active inhibitory form, e*. The e* inhibitor diffuses away from kinetochores and binds to the target **c**, the protein required for cell cycle progression. This creates, through an irreversible step, the inhibited form c*, which can then decay spontaneously and irreversibly into **c** and **e**. Although Barkai and collaborators [17] did not define the molecular identities of **e**, e*, **c**, and c*, we propose that **e** is O-Mad2, **c** is free Cdc20, e* is the active form of Mad2 that binds Cdc20, and c* is the inhibited form of Cdc20 (i.e., bound to C-Mad2).

There are two main differences between the emitted inhibition model and the Mad2 template model. The first difference concerns the presence of an autocatalytic loop in the Mad2 template model. The autocatalytic loop is based on the ability of Cdc20-bound C-Mad2 to help the conversion of O-Mad2, and it stems naturally from the structural similarities between the Mad1-bound and the Cdc20-bound C-Mad2. Previously, the existence of such an autocatalytic loop was ruled out based on the notion that it would instate a steady state with an operational SAC from which cells could not escape [17]. We argue that this effect was an inevitable consequence of the (unproven) assumption that the formation of the inhibitory complex is irreversible. Thus, a second difference between our model and the emitted inhibition model (Figure 6B) is that in our model, the hypothesized catalytic reactions (autocatalysis and catalysis, reactions 3 and 5 in Figure 1D) do not require any energy sources, and thus in the system analyzed here, they do not create or affect the steady state of the binding reaction between Mad2 and Cdc20. All reactions are fully reversible and indeed are expected to drive the system towards a state in which a large fraction of Cdc20 is bound to Mad2 (Figure 6A), but that is primarily due to the low *KD*_bind_. The steady-state Mad2-bound Cdc20 would be basically the same even in the absence of the catalytic reactions (i.e., only in presence of reaction 1 in Figure 1D)

This feature of the SAC might explain the ability of cells to maintain the SAC for very long times (∼24 h in human cells), and up to several days in some cases. At the same time, it poses the problem of how the SAC can be switched off in vivo. We predict, in agreement with the emitted inhibition model, that this occurs via an energy-driven process that creates a new steady state with SAC OFF (high concentration of free Cdc20). Most likely, the new steady state is achieved by inducing the energy-dependent release of Cdc20 from the complex Cdc20:C-Mad2. Recent data showing that Cdc20 ubiquitination (i.e., an ATP-dependent process) is required to free Cdc20 from bound Mad2 support our hypothesis [37,38].

If we recast the Mad2 template model using the formalism adopted by Barkai and colleagues [17], we end up with the scheme shown in Figure 6B. Here, catalysis simply accelerates the completion of the reaction, as schematized in Figure 6C, and all reactions are reversible, except for the dissociation of the inhibited complex c*, which reflects the above-mentioned energy-dependent reaction that releases Cdc20.

### An Energetic Point of View of the SAC

The maintenance of a SAC OFF state—in which the Cdc20:C-Mad2 complex cannot accumulate—might be rather cheap from the energetic standpoint. This is because the very large activation energy built into the Mad2 conversion works to maintain Mad2 in the O-Mad2 conformation, at least until O-Mad2 is allowed to dimerize with C-Mad2 (Figure 6C). In this scenario, the fact that the initial, noncatalysed association and dissociation rates are very slow guarantees the possibility to prevent the accumulation of Cdc20:C-Mad2 complexes. The proposed energy-dependent mechanism of Cdc20:C-Mad2 dissociation might contribute to the removal of Cdc20:C-Mad2 complexes that might form, slowly but spontaneously, in cell cycle phases other than M phase.

The need of a tight Mad1:C-Mad2 complex at the start of mitosis, when the checkpoint needs to be activated, fits nicely into this picture. Mad1:C-Mad2 allows the rapid initial rate of Mad2 binding to Cdc20, and avoids wasting time for the slow step of the reaction, the conversion from O-Mad2 to C-Mad2. The rapid formation of Cdc20:C-Mad2 triggered by conformational dimerization and other mechanisms exceeds the ability of the energy dependent mechanisms to continuously create free Cdc20, leading to the accumulation of Cdc20:C-Mad2. Previous analyses have suggested that the activation of Mad1:C-Mad2 early in prometaphase might require the inactivation of p31^comet^, a negative regulator of the SAC that acts by binding to C-Mad2 in the Mad1:C-Mad2 complex, thus preventing its interaction with O-Mad2 [21,29,30]. The molecular details of p31^comet^ temporary inactivation, however, remain unclear.

The role of Mad1:C-Mad2 in SAC activation is illustrated with a simulation of a hypothetical system in which Mad1 binds to kinetochores as a monomer, and only later it recruits O-Mad2 to form the Mad1:C-Mad2 complex (Figure 6A, green trace). Because the noncatalysed binding of O-Mad2 to Cdc20 is very slow, the binding of O-Mad2 to Mad1, which implicates the same conformational change of Mad2, is expected to be equally slow. Even if Mad1:C-Mad2 were endowed with increased catalytic power (e.g., 300-fold additional acceleration), its contribution would be very significantly delayed relative to that achieved with preformed Mad1:C-Mad2 (Figure 6A, orange trace. The *t*_1/2_ would be 175 min and 10 min, respectively).

The formation of Cdc20:C-Mad2, in turn, is likely to result in the formation of a larger inhibitory complex (the MCC) with the Bub3:BubR1/Mad3 complex. Although this paper focuses on what is possibly the first step in MCC formation, hereafter we provide a speculative overview of the series of reactions that lead from the isolated components to the formation of the MCC (Figure 6C). Previous analyses have shown that the presence of Mad2 favours the formation of a Bub3:BubR1/Mad3 complex in vitro and in vivo [5,40–45], even if a Cdc20:Bub3:BubR1/Mad3 complex can be formed from purified components in vitro [43,44]. Conversely, to our knowledge, there is little or no evidence that BubR1/Mad3 is necessary for assembling a complex of Mad2 with Cdc20. The requirements on Mad2 for the incorporation of Bub3:BubR1/Mad3 in the MCC is reminiscent of the requirement of Mad1:C-Mad2 for the formation of the Cdc20:C-Mad2 complex. It leads us to speculate that the network of interactions that are responsible for SAC activation might consist of two consecutive kinetically limited steps. In this scheme, the initial activation of Mad2 will allow Bub3:BubR1/Mad3 to bind Cdc20 and the APC/C as shown in Figure 6C. The diagram in Figure 6C clarifies that the formation of the MCC might not require external energy sources, but it requires catalysis. In the absence of catalysts, a checkpoint defect will arise due to the rapid APC/C-mediated destruction of cyclin B and securin, which will drive cells out of mitosis. This model provides a molecular network to the hypothesis that kinetochores act as catalysts in the formation of an anaphase inhibitor [46]. The autocatalytic loop supposedly active in the cytoplasm could have an important role to guarantee a robust SAC with a small number of unattached kinetochores.

In conclusion, we provided evidence that catalysis drives the formation of Cdc20:Mad2 complexes as initially suggested by the Mad2-template model. We found that catalysis not only is possible, but is indeed required for an efficient and functional checkpoint. Indeed, our studies show that although the catalytic conversion of O-Mad2 based on its dimerization with C-Mad2 might be necessary to accelerate O-Mad2 binding to Cdc20, it is unlikely to be sufficient. Future studies will have to concentrate on the important problem of identifying by which additional mechanisms, besides Mad2 dimerization, the interaction of Mad2 with Cdc20 can be accelerated.

## Materials and Methods

### Protein purification.

Full-length Mad2^wt^, Mad2^F141A^, and p31^comet^ were purified as described [14] with the exception of the final size-exclusion chromatography separation, which was performed on a Superdex-75 column (GE Healthcare) equilibrated in PBS buffer. Biotinylated Mad1:C-Mad2 was expressed and purified using an AviTag system (Avidity) as described [28].

### Labelling with Alexa 488.

The 100 μM purified Mad2^wt^ and Mad2^F141A^ were incubated at 4 °C for 12 h with a 10-fold molar excess of Alexa 488 C_5_ maleimide (Molecular Probes) and 2 mM tris(2-carboxyethyl)phosphine (TCEP) in PBS. Size-exclusion chromatography on a Superdex-75 column was used to separate the unbound fraction of dye from labelled proteins.

### Kinetics experiments.

Ten-microlitre chambers (μ-slide V; Ibidi) were coated with neutravidin. Chambers were equilibrated with 150 μl of MilliQ water and 15 μl of neutravidin 1 mg/ml (A-2666; Molecular Probes) in TRIS-EDTA buffer. A total of 15 μl of 10 mg/ml BSA in MilliQ water was added step by step with 30-min incubations followed by 150 μl of MilliQ washing. After equilibration with 150 μl of PBS, 15 μl of synthetic biotin-Cdc20^111−138^ at a concentration of 25 μM in PBS was added to the chamber and incubated for 30 min. Finally, the chambers were washed with 150 μl of PBS. The time courses were performed by injecting Alexa-488-Mad2^wt^ or Alexa-488-Mad2^F141A^ at concentrations in the 1–8 μM range. Fluorescence localization on the surface was monitored on a Leica Microsystems TCS SP2 confocal microscope equipped with a 63×/1.40 (OIL CS HC3PL APO) objective lens. Imaging was controlled by Leica Confocal Software (v. 2.61), and acquisition was carried out with the 488-nm line of an Ar/ArKr laser. ImageJ (National Institutes of Health, http://rsb.info.nih.gov/ij/, 1997–2004) and Omogen software programs were used to calculate mean pixel intensities at the surface. Omogen is a software developed by the authors and available upon request.

### “Doping” the surface with preformed C-Mad2.

Purified biotinylated Mad1:C-Mad2 complex was immobilized onto the surface of the μ-slide V. The surface was then coated with neutravidin as described above. After equilibration with 150 μl of PBS, 15 μl of 0.250 μM biotinylated Mad1:C-Mad2 complex in PBS and 15 μl of 25 μM synthetic biotin-Cdc20 peptide^111−138^ in PBS were added and incubated for 30 min. The surface was then washed with 150 μl of PBS.

### Parameters estimation.

Parameter estimation was performed with PET, a free software developed by Dr. Jason Zwolak (Virginia Polytechnic Institute and State University). Numerical simulations were carried out with XPP-AUT, a free software program developed by Prof. Bard Ermentrout (Department of Mathematics, University of Pittsburgh; http://rd.plos.org/pbio.1000010) Additional details on parameter estimation are to be found in Text S1.

## Supporting Information

Figure S1 [Cdc20] at Equilibrium as a Function of *KD*_bind_
In Figure 2C, we aim to verify experimentally that Mad2^wt^ and Mad2^F141A^ share the same *KD*_bind_ by measuring the amount of Cdc20 that they bind at equilibrium. Here, we provide evidence for the assumption that if the two proteins bind a similar amount of Cdc20, then they must have a similar *KD*_bind_. We plot [Cdc20] at equilibrium as a function of *KD*_bind_, both for Mad2^F141A^ (black line; reaction 1 in Figure 1D) and Mad2^wt^ (green line; this being an equilibrium analysis, we only take into account reactions 1 and 4 in Figure 1D). We show that if *KD*_dim_ is 1.5 μM, compatible with the values reported in [28], Mad2^F141A^ and Mad2^wt^ are expected to bind a very similar amount of Cdc20 for a wide range of *KD*_bind_. The relative amounts of [Cdc20] at equilibrium for Mad2^wt^ and for Mad2^F141A^ with *KD*_bind_ 0.1 μM (the value estimated in this paper) are marked by a green-filled dot and a black-filled dot, respectively. As in the experimental setting for Figure 2C, [Cdc20_T_] = 1 μM, and [Mad2_T_] = 1 μM.
(25 KB PDF)

Figure S2 Binding of Mad2 to Cdc20 as Followed in Solution Rather Than on the Surface Left, the mutant Mad2^F141A^. Simulations (black curves) produced with reaction 1 in Table S1 and the following parameters: *KD*_bind_ = 0.24 μM, k_bind,on_ = 2.7 × 10^−5^ μM^−1^ s^−1^, [Cdc20_T_] = 0.9 μM. Right, Mad2^wt^. Simulations (black curves) produced with reactions 1, 4, and 5 in Table S1, the same parameters as for the Mad2^F141A^, plus *KD*_dim_ = 2.45 μM, *k*_dim,on_ = 0.7 μM^−1^ s^−1^, *KD*_cat_ = 0.07, and *k*_cat,on_ = 0.00175 μM^−1^ s^−1^. Experimental results (coloured dots) differ for [Mad2^wt^_T_] or [Mad2^F141A^_T_], which can be read at time zero on the *y*-axis. Similar colours represent different time series for the same amount of [Mad2_T_]. The experimental curves for [Mad2^F141A^_T_] = 8 μM and [Mad2^wt^_T_] = 2 and 3 μM are shown until the time point after which the constant decrease contributes to more than 0.2 μM of the signal (see Text S1 for details).
(84 KB PDF)

Figure S3 Predictions of the Model from Analysis of Fluorescence in Solution The model predicts that the binding of Cdc20 and Mad2 should be accelerated in presence of Mad1:C-Mad2 bound onto the surface, and should be delayed in presence of p31^comet^. Here, we plot the predictions and experimental data as obtained from the signal in solution. Black solid lines: predictions (equations in Tables S1 and S2, parameters in Figure S2 and Table 1); dots are experimental data. Red dots: 2 μM Mad2^wt^ was added in solution together with 10 μM p31^comet^. Dark- and light-blue dots: two different time courses for 2 μM Mad2^wt^. Green and yellow dots: two time courses of 2 μM Mad2^wt^, in presence of an estimated 0.25 μM Mad1:C-Mad2 bound onto the surface.
(47 KB PDF)

Figure S4 Determination of Cdc20 Bound onto the Surface We performed an experiment to estimate the amount of Cdc20 bound onto the surface, expressed as the number of moles bound over the volume of the chamber in litres. Alexa-545-Cdc20 binds both to avidin (i.e., specifically) as well as to unidentified receptors on the surface (i.e., aspecifically). To distinguish between these two populations, experiments are performed either adding Alexa-545-Cdc20 directly, or adding it after pretreating the chamber with biotin. In the first case, both aspecific and specific binding will occur, whereas in the second, the binding will only be aspecific since no avidin sites will be available for Alexa-545-Cdc20. First, we estimated the saturating amount of Cdc20, i.e., the minimal concentration of Cdc20 in the volume of the chamber that will saturate all the binding sites, and then we set to measure the amount of Cdc20 that actually binds onto the surface.
(A) Determination of the saturating concentration of Cdc20. The chambers are coated as described in Materials and Methods and then treated or not with a large excess of biotin. Alexa-545-Cdc20 is added to the chambers and incubated for 30 min to allow a complete binding. Unbound proteins are washed away, and fluorescence on the surface is measured at equilibrium. Experiments are performed with five different concentrations of Alexa-545-Cdc20 (25 μM, 10 μM, 5 μM, 1 μM, and 0.5 μM). For each concentration of Cdc20, fluorescence on the surface at steady state is plotted in presence (blue) or absence (red) of biotin pretreatment. The saturating amount of Cdc20 can be estimated between 1 and 2 μM.
(B) The specific binding of Cdc20 is calculated as the difference between the fluorescence measured on the surface without and with biotin pretreatment. Again, the saturating amount of Cdc20 can be estimated between 1 and 2 μM.
(C) Estimate of Cdc20 bound onto the surface. The chambers are coated as described in Materials and Methods and then treated (blue), or not treated (red), with a large excess of biotin. A saturating amount of Alexa-545-Cdc20 (2 μM) is then added to the chambers. Binding can be measured as the difference between the initial concentration in solution (2 μM) and the remaining concentration at equilibrium. This amounts to 1 μM Cdc20 for the total binding (red curve) of which 0.5 μM is due to aspecific binding (blue curve).
(38 KB PDF)

Figure S5 Residuals of the Fitting (A) Mutant Mad2^F141A^. For each time point of the series in Figure 3D, we plot the difference between experimental values and fitted curve.
(B) The same analysis is shown for Mad2^wt^ (experimental and fitted data in Figure 4B).
(88 KB PDF)

Table S1 Differential and Algebraic Equations Differential equations have been derived by applying the law of mass action to the reactions in Figure 1D, as reported in the names of the different rates. In this table, we present a system of ordinary differential equations (ODEs) that includes all the molecular species; but in different simulations, a smaller subset of reactions is included, as explained in Table S2. The resulting systems of ODEs and algebraic equations can be derived accordingly.
(246 KB PDF)

Table S2 Combinations of Reactions Used for Simulations The simulations that we report in the paper are obtained by differential equations that are produced by different combinations of the reactions enlisted in Table S1. Here, we report the reactions that have been used for each simulation. The conservation relations can be deduced accordingly.
(40 KB PDF)

Text S1 Methods Used for Parameter Estimation (150KB PDF).

## Abbreviations

APC/C: anaphase-promoting complex/cyclosome
C-Mad2: closed Mad2
GST: glutathione S-transferase
I-Mad2: intermediate Mad2
MCC: mitotic checkpoint complex
NEB: nuclear envelope breakdown
O-Mad2: open Mad2
SAC: spindle assembly checkpoint

## References

[pbio-1000010-b001] Nasmyth K (1999). Separating sister chromatids. Trends Biochem Sci.

[pbio-1000010-b002] Musacchio A, Salmon ED (2007). The spindle-assembly checkpoint in space and time. Nat Rev Mol Cell Biol.

[pbio-1000010-b003] Peters JM (2006). The anaphase promoting complex/cyclosome: a machine designed to destroy. Nat Rev Mol Cell Biol.

[pbio-1000010-b004] King EM, van der Sar SJ, Hardwick KG (2007). Mad3 KEN boxes mediate both Cdc20 and Mad3 turnover, and are critical for the spindle checkpoint. PLoS ONE.

[pbio-1000010-b005] Burton JL, Solomon MJ (2007). Mad3p, a pseudosubstrate inhibitor of APCCdc20 in the spindle assembly checkpoint. Genes Dev.

[pbio-1000010-b006] Cheeseman IM, Desai A (2008). Molecular architecture of the kinetochore-microtubule interface. Nat Rev Mol Cell Biol.

[pbio-1000010-b007] Rieder CL, Cole RW, Khodjakov A, Sluder G (1995). The checkpoint delaying anaphase in response to chromosome monoorientation is mediated by an inhibitory signal produced by unattached kinetochores. J Cell Biol.

[pbio-1000010-b008] Mapelli M, Musacchio A (2007). MAD contortions: conformational dimerization boosts spindle checkpoint signaling. Curr Opin Struct Biol.

[pbio-1000010-b009] Luo X, Tang Z, Rizo J, Yu H (2002). The Mad2 spindle checkpoint protein undergoes similar major conformational changes upon binding to either Mad1 or Cdc20. Mol Cell.

[pbio-1000010-b010] Luo X, Tang Z, Xia G, Wassmann K, Matsumoto T (2004). The Mad2 spindle checkpoint protein has two distinct natively folded states. Nat Struct Mol Biol.

[pbio-1000010-b011] Chung E, Chen R-H (2002). Spindle checkpoint requires Mad1-bound and Mad1-free Mad2. Mol Biol Cell.

[pbio-1000010-b012] Shah JV, Botvinick E, Bonday Z, Furnari F, Berns M (2004). Dynamics of centromere and kinetochore proteins; implications for checkpoint signaling and silencing. Curr Biol.

[pbio-1000010-b013] De Antoni A, Pearson CG, Cimini D, Canman JC, Sala V (2005). The mad1/mad2 complex as a template for mad2 activation in the spindle assembly checkpoint. Curr Biol.

[pbio-1000010-b014] Mapelli M, Massimiliano L, Santaguida S, Musacchio A (2007). The Mad2 conformational dimer: structure and implications for the spindle assembly checkpoint. Cell.

[pbio-1000010-b015] Nezi L, Rancati G, De Antoni A, Pasqualato S, Piatti S (2006). Accumulation of Mad2-Cdc20 complex during spindle checkpoint activation requires binding of open and closed conformers of Mad2 in Saccharomyces cerevisiae. J Cell Biol.

[pbio-1000010-b016] Yang M, Li B, Liu CJ, Tomchick DR, Machius M (2008). Insights into mad2 regulation in the spindle checkpoint revealed by the crystal structure of the symmetric mad2 dimer. PLoS Biol.

[pbio-1000010-b017] Doncic A, Ben-Jacob E, Barkai N (2005). Evaluating putative mechanisms of the mitotic spindle checkpoint. Proc Natl Acad Sci U S A.

[pbio-1000010-b018] Sear RP, Howard M (2006). Modeling dual pathways for the metazoan spindle assembly checkpoint. Proc Natl Acad Sci U S A.

[pbio-1000010-b019] Ibrahim B, Dittrich P, Diekmann S, Schmitt E (2008). Mad2 binding is not sufficient for complete Cdc20 sequestering in mitotic transition control (an in silico study). Biophys Chem.

[pbio-1000010-b020] Sironi L, Melixetian M, Faretta M, Prosperini E, Helin K (2001). Mad2 binding to Mad1 and Cdc20, rather than oligomerization, is required for the spindle checkpoint. EMBO J.

[pbio-1000010-b021] Mapelli M, Filipp FV, Rancati G, Massimiliano L, Nezi L (2006). Determinants of conformational dimerization of Mad2 and its inhibition by p31(comet). EMBO J.

[pbio-1000010-b022] Sironi L, Mapelli M, Knapp S, Antoni AD, Jeang K-T (2002). Crystal structure of the tetrameric Mad1-Mad2 core complex: implications of a ‘safety belt' binding mechanism for the spindle checkpoint. EMBO J.

[pbio-1000010-b023] Tang Z, Shu H, Oncel D, Chen S, Yu H (2004). Phosphorylation of Cdc20 by Bub1 provides a catalytic mechanism for APC/C inhibition by the spindle checkpoint. Mol Cell.

[pbio-1000010-b024] D'Angiolella V, Mari C, Nocera D, Rametti L, Grieco D (2003). The spindle checkpoint requires cyclin-dependent kinase activity. Genes Dev.

[pbio-1000010-b025] Chung E, Chen RH (2003). Phosphorylation of Cdc20 is required for its inhibition by the spindle checkpoint. Nat Cell Biol.

[pbio-1000010-b026] Kramer ER, Scheuringer N, Podtelejnikov AV, Mann M, Peters JM (2000). Mitotic regulation of the APC activator proteins CDC20 and CDH1. Mol Biol Cell.

[pbio-1000010-b027] Yudkovsky Y, Shteinberg M, Listovsky T, Brandeis M, Hershko A (2000). Phosphorylation of Cdc20/fizzy negatively regulates the mammalian cyclosome/APC in the mitotic checkpoint. Biochem Biophys Res Commun.

[pbio-1000010-b028] Vink M, Simonetta M, Transidico P, Ferrari K, Mapelli M (2006). In vitro FRAP identifies the minimal requirements for Mad2 kinetochore dynamics. Curr Biol.

[pbio-1000010-b029] Xia G, Luo X, Habu T, Rizo J, Matsumoto T (2004). Conformation-specific binding of p31(comet) antagonizes the function of Mad2 in the spindle checkpoint. EMBO J.

[pbio-1000010-b030] Yang M, Li B, Tomchick DR, Machius M, Rizo J (2007). p31comet blocks Mad2 activation through structural mimicry. Cell.

[pbio-1000010-b031] Meraldi P, Draviam VM, Sorger PK (2004). Timing and checkpoints in the regulation of mitotic progression. Dev Cell.

[pbio-1000010-b032] Geley S, Kramer E, Gieffers C, Gannon J, Peters JM (2001). Anaphase-promoting complex/cyclosome-dependent proteolysis of human cyclin A starts at the beginning of mitosis and is not subject to the spindle assembly checkpoint. J Cell Biol.

[pbio-1000010-b033] den Elzen N, Pines J (2001). Cyclin A is destroyed in prometaphase and can delay chromosome alignment and anaphase. J Cell Biol.

[pbio-1000010-b034] Howell BJ, Hoffman DB, Fang G, Murray AW, Salmon ED (2000). Visualization of Mad2 dynamics at kinetochores, along spindle fibers, and at spindle poles in living cells. J Cell Biol.

[pbio-1000010-b035] Howell BJ, Moree B, Farrar EM, Stewart S, Fang G (2004). Spindle checkpoint protein dynamics at kinetochores in living cells. Curr Biol.

[pbio-1000010-b036] DeAntoni A, Sala V, Musacchio A (2005). Explaining the oligomerization properties of the spindle assembly checkpoint protein Mad2. Philos Trans R Soc Lond B Biol Sci.

[pbio-1000010-b037] Reddy SK, Rape M, Margansky WA, Kirschner MW (2007). Ubiquitination by the anaphase-promoting complex drives spindle checkpoint inactivation. Nature.

[pbio-1000010-b038] Stegmeier F, Rape M, Draviam VM, Nalepa G, Sowa ME (2007). Anaphase initiation is regulated by antagonistic ubiquitination and deubiquitination activities. Nature.

[pbio-1000010-b039] Luo X, Fang G, Coldiron M, Lin Y, Yu H (2000). Structure of the mad2 spindle assembly checkpoint protein and its interaction with cdc20. Nat Struct Biol.

[pbio-1000010-b040] Hwang LH, Lau LF, Smith DL, Mistrot CA, Hardwick KG (1998). Budding yeast Cdc20: a target of the spindle checkpoint. Science.

[pbio-1000010-b041] Davenport J, Harris LD, Goorha R (2006). Spindle checkpoint function requires Mad2-dependent Cdc20 binding to the Mad3 homology domain of BubR1. Exp Cell Res.

[pbio-1000010-b042] Chen R-H (2002). BubR1 is essential for kinetochore localization of other spindle checkpoint proteins and its phosphorylation requires Mad1. J Cell Biol.

[pbio-1000010-b043] Fang G (2002). Checkpoint protein BubR1 acts synergistically with Mad2 to inhibit anaphase-promoting complex. Mol Biol Cell.

[pbio-1000010-b044] Tang Z, Bharadwaj R, Li B, Yu H (2001). Mad2-independent inhibition of APC-Cdc20 by the mitotic checkpoint protein Bub1R. Dev Cell.

[pbio-1000010-b045] Hardwick KG, Johnston RC, Smith DL, Murray AW (2000). MAD3 encodes a novel component of the spindle checkpoint which interacts with Bub3p, Cdc20p, and Mad2p. J Cell Biol.

[pbio-1000010-b046] Gorbsky GJ, Kallio M, Daum JR, Topper LM (1999). Protein dynamics at the kinetochore: cell cycle regulation of the metaphase to anaphase transition. FASEB J 13 Suppl.

[pbio-1000010-b047] Habu T, Kim SH, Weinstein J, Matsumoto T (2002). Identification of a MAD2-binding protein, CMT2, and its role in mitosis. EMBO J.

