# Supplementary material for: The Influence of Catalysis on Mad2 Activation Dynamics
Source: PLoS Biol. 2009 Jan 13;7(1):e1000010. doi: 10.1371/journal.pbio.1000010 (PMC2621267; doi:10.1371/journal.pbio.1000010)
Supplement: Supplementary file 1 [file pbio.1000010.sd001.pdf]

Role of diffusion and space Fitting was performed using the ordinary differential equations (ODEs) in Table S1. The ODEs implicitly assume that both receptors (Cdc20) and ligands (Mad2) are free to move in solution. In reality, we immobilized Cdc20 onto the surface of the chamber. This gives rise to two important concerns regarding diffusion and catalysis, which we address hereafter.

Role of Diffusion Before applying systems of ODEs to fit the experimental data, we had to make sure that the reactions under consideration are reaction-limited and not diffusion-limited. Following [1], the component of the reaction rate due to diffusion can be expressed as $\mathrm{k}_{diff} = 4 \cdot \pi \cdot D \cdot s$, where D is the diffusion coefficient and s the radius of the molecule. Given that the radius of Mad2 (a protein of 25 kDa) is approximately 30 Å and choosing a conservative estimate for the diffusion coefficient in water (20 $\mu m^2 sec^{-1}$) [2], we find $k_{\mathrm{diff}} \approx 400\ \mu M^{-1}\ sec^{-1}$. This value is orders of magnitude larger than any rate constant measured in our system, thus ruling out a major role for diffusion.

Catalysis on the surface The use of ODEs to describe reactions 3 and 5 in Figure 1D implies that a molecule of Cdc20:C-Mad2:O-Mad2 (or Mad1:C-Mad2:O-Mad2) can donate the primed, external O-Mad2 to every molecule of Cdc20. In fact, in our experimental setting the molecules of Cdc20 are bound onto the surface, where catalysis is expected to occur. Therefore, only the molecules of Cdc20 that are nearest neighbours of Cdc20:C-Mad2:O-Mad2 (or Mad1:C-Mad2:O-Mad2) will be able to accept molecules of primed O-Mad2, if this latter is short-lived. This inconsistency between the experimental set-up and our modeling approach might explain why the fitting that we obtain is not perfect, albeit very accurate (Figure 3D, Figure 4B and Figure S5). Better fitting could be produced with a model that includes the spatial dimension, which would

result in a level of sophistication beyond the aim of this paper. We rather opted for the ODEs, arguably the simplest possible tool, which, regardless of its limitations, gives quite a good fitting.

Parameter estimation The binding reaction can be followed either via the disappearance of the Mad2 fluorescence signal in solution, or through the increase of fluorescence on the surface. As an internal check, we decided to analyze both of them. In the main text, we present the fitting for the signal on the surface (Figures 3 and 4). Here we give the technical details for parameter estimation, while the results of fitting the signal in solution are shown in Figure S2.

Parameter estimation for the signal measured on the surface The confocal images were processed by a software developed for this paper, Omogen, that quantifies the amount of signal accumulated on the surface during the experiment (Omogen is available upon request). Each point in the time-series was subtracted of the background signal present on the surface at time zero and was divided by the average over the last 10% points, provided that the series had reached a plateau. Global fitting was performed with PET (freeware developed by Jason Zwolak [3], http://mpf.biol.vt.edu/pet/), using the differential equations in Table S1 normalized by the plateau value of fluorescence on the surface computed as [Cdc20:C-Mad2]+2[Cdc20:C-Mad2:O-Mad2]. All available data sets were simultaneously fitted by the program.

Parameter values were searched within limits dictated by available data. The limits for $[Cdc20_T]$ were set to 0.5 and 1 μM based on the experiment described in Figure S4. The upper limit for the dissociation constant of the basal rate (reaction 1 in Table S1) is

$k_{\text{bind,off}}/k_{\text{bind,on}}$ = 1 μM. As for $k_{\text{bind,on}}$, we set 10^{-4} and 10^{-6} μM^{-1} sec^{-1} as upper and lower limits, respectively. Global fitting of the data obtained with $\text{Mad2}^{\text{F141A}}$ returned the values for $k_{\text{bind,on}}$, KD_{bind} and [Cdc20_{T}] reported in Table I. These values were then used for the parameter estimation of Mad2^{wt} data, that lead to the estimation of the remaining parameter, $k_{\text{cat,on}}$. The values of $k_{\text{dim,on}}$ and $k_{\text{dim,off}}$ have been published recently [4]. The residuals (Figure S5) show the presence of trends in the deviation between the experimental curve and our fitting during the early stages of binding. On the other hand, it should be remarked that fitting was done changing one parameter only, and all experimental curves were simultaneously fitted with the very same parameters. The goodness of fit emerges clearly in the predictions, Figure 5B, 5D and 5G, that are quantitatively in agreement with the experimental data.

Parameter estimation for the signal measured in solution The signal in solution was processed with Omogen. The raw data were subtracted of the signal present in the chamber in the absence of Mad2. The conversion factor between fluorescence and concentration was computed by dividing [Mad2_{T}] by the signal detected in solution in the first image. Every experimental value was then multiplied by this factor, to obtain the time course of [Mad2]. Compared to the data obtained on the surface, the signal showed a constant decrease, partially independent from the binding of Mad2 to Cdc20. No Mad2 accumulated onto the surface when Cdc20 was replaced by a scrambled peptide unable to bind Mad2 but with the same physico-chemical properties of Cdc20. Nevertheless, the signal in solution was decreasing according to an exponential decay $\frac{d[\text{Mad2}]}{dt} = [\text{Mad2}_{\text{T}}]e^{-k_{decay} \cdot t}$ with $k_{\text{decay}} = 2.5 \cdot 10^{-4}$ sec^{-1} for $\text{Mad2}^{\text{F141A}}$ and $1.7 \cdot 10^{-3} sec^{-1}$ for Mad2^{wt} (not shown). Given these values, at the beginning of the reaction the loss of

signal in solution is mainly due to the binding of Mad2 to Cdc20. To make sure not to fit this additional loss of signal, we limited the fitting up to the time point when it contributes to more than 0.2 μM to the loss of signal from the solution. Parameter estimation gave values of association, dimerization and catalysis compatible with those obtained on the surface, as shown in Figure S2.

References

1. Shoup D, Szabo A (1982) Role of diffusion in ligand binding to macromolecules and cell-bound receptors. Biophys J 40: 33-39.
2. Lauffenburger DA, Linderman JJ (1993) Receptors: Oxford University Press. p. 143-152
3. Zwolak JW, Tyson JJ, Watson LT (2005) Parameter estimation for a mathematical model of the cell cycle in frog eggs. J Comput Biol 12: 48-63.
4. Vink M, Simonetta M, Transidico P, Ferrari K, Mapelli M, et al. (2006) In Vitro FRAP Identifies the Minimal Requirements for Mad2 Kinetochore Dynamics. Curr Biol 16: 755-766.
