# Supplementary material for: The Influence of Catalysis on Mad2 Activation Dynamics
Source: PLoS Biol. 2009 Jan 13;7(1):e1000010. doi: 10.1371/journal.pbio.1000010 (PMC2621267; doi:10.1371/journal.pbio.1000010)
Supplement: Supplementary file 2 [file pbio.1000010.sg001.pdf]

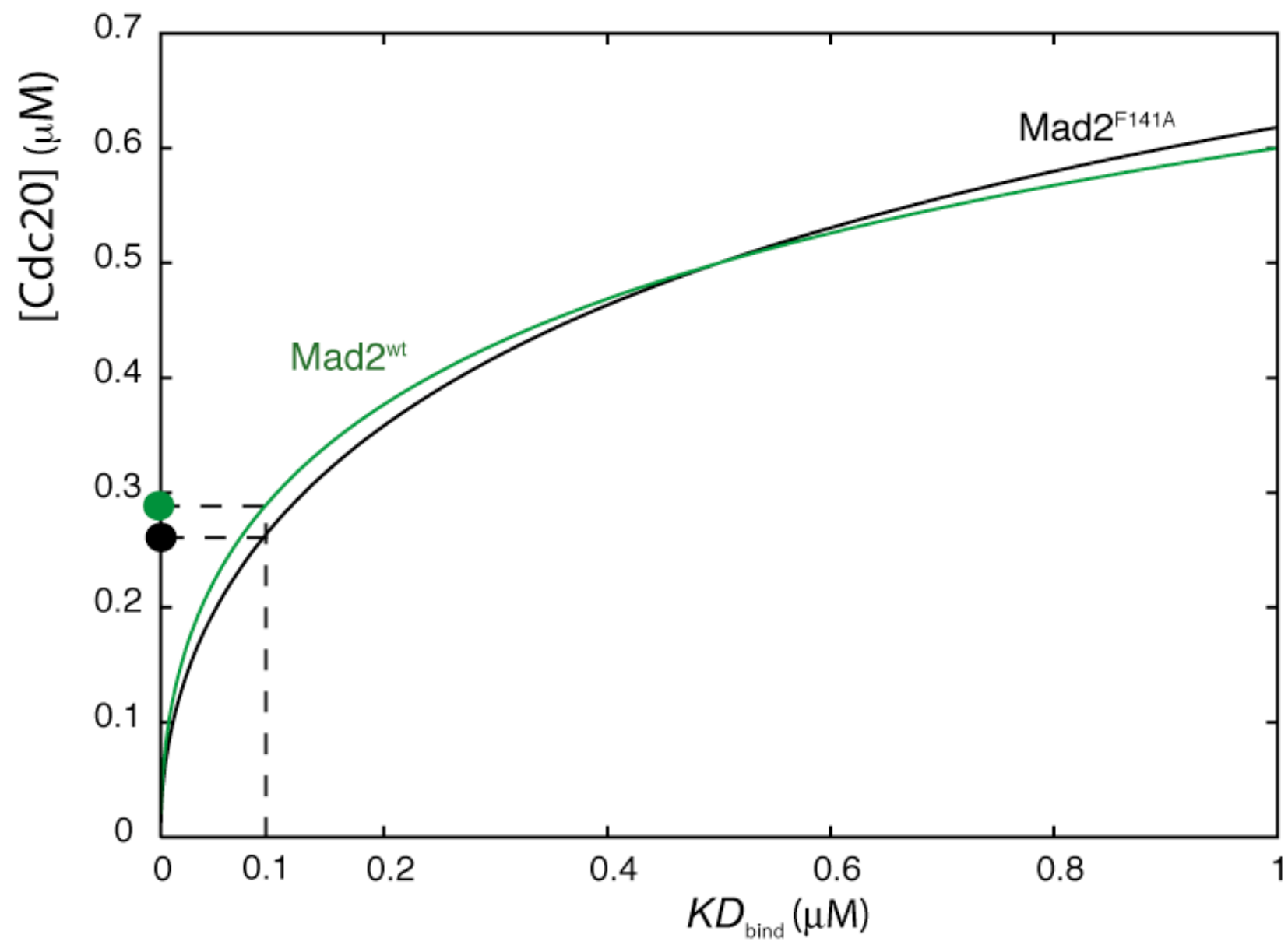

[Cdc20] (μM)
0.7
0.6
0.5
0.4
0.3
0.2
0.1
0
Mad2F141A
Mad2wt
0
0.1
0.2
0.4
0.6
0.8
1
KDbind (μM)
