# Supplementary material for: The Influence of Catalysis on Mad2 Activation Dynamics
Source: PLoS Biol. 2009 Jan 13;7(1):e1000010. doi: 10.1371/journal.pbio.1000010 (PMC2621267; doi:10.1371/journal.pbio.1000010)
Supplement: Supplementary file 3 [file pbio.1000010.sg002.pdf]

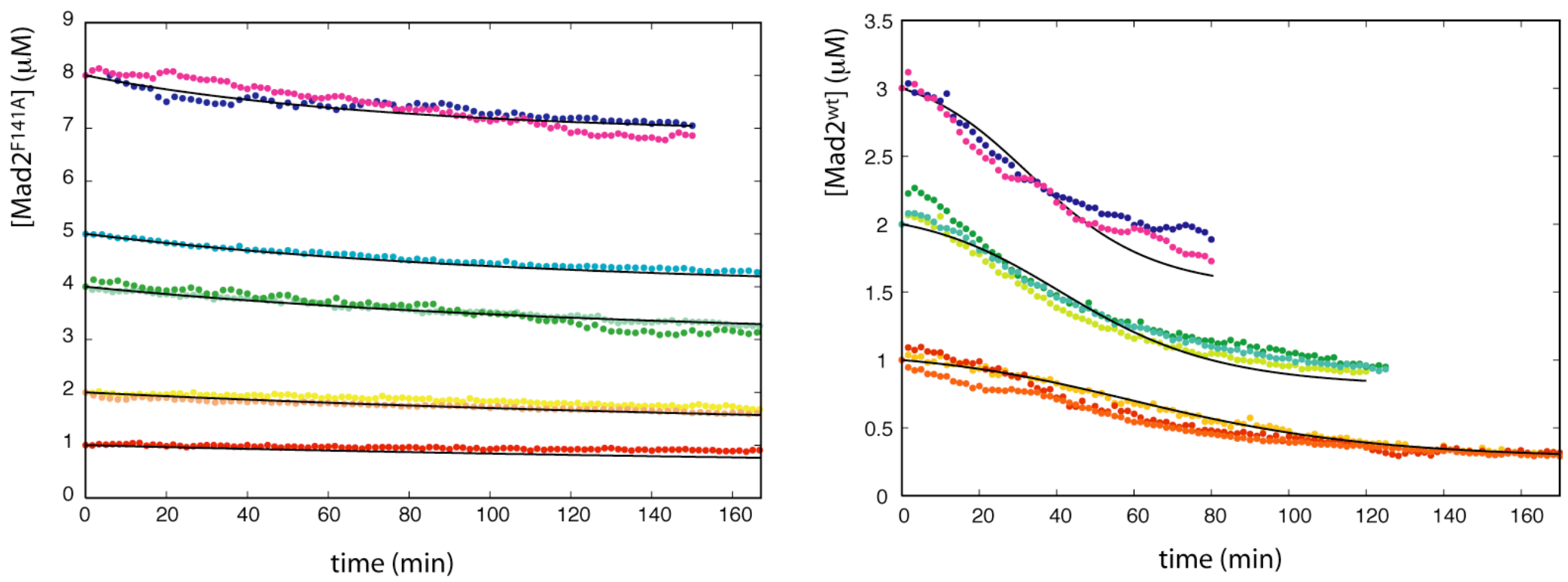

[Mad2^{F141A}] (μM)
time (min)
[Mad2^{wt}] (μM)
time (min)
