# Supplementary material for: The Influence of Catalysis on Mad2 Activation Dynamics
Source: PLoS Biol. 2009 Jan 13;7(1):e1000010. doi: 10.1371/journal.pbio.1000010 (PMC2621267; doi:10.1371/journal.pbio.1000010)
Supplement: Supplementary file 4 [file pbio.1000010.sg003.pdf]

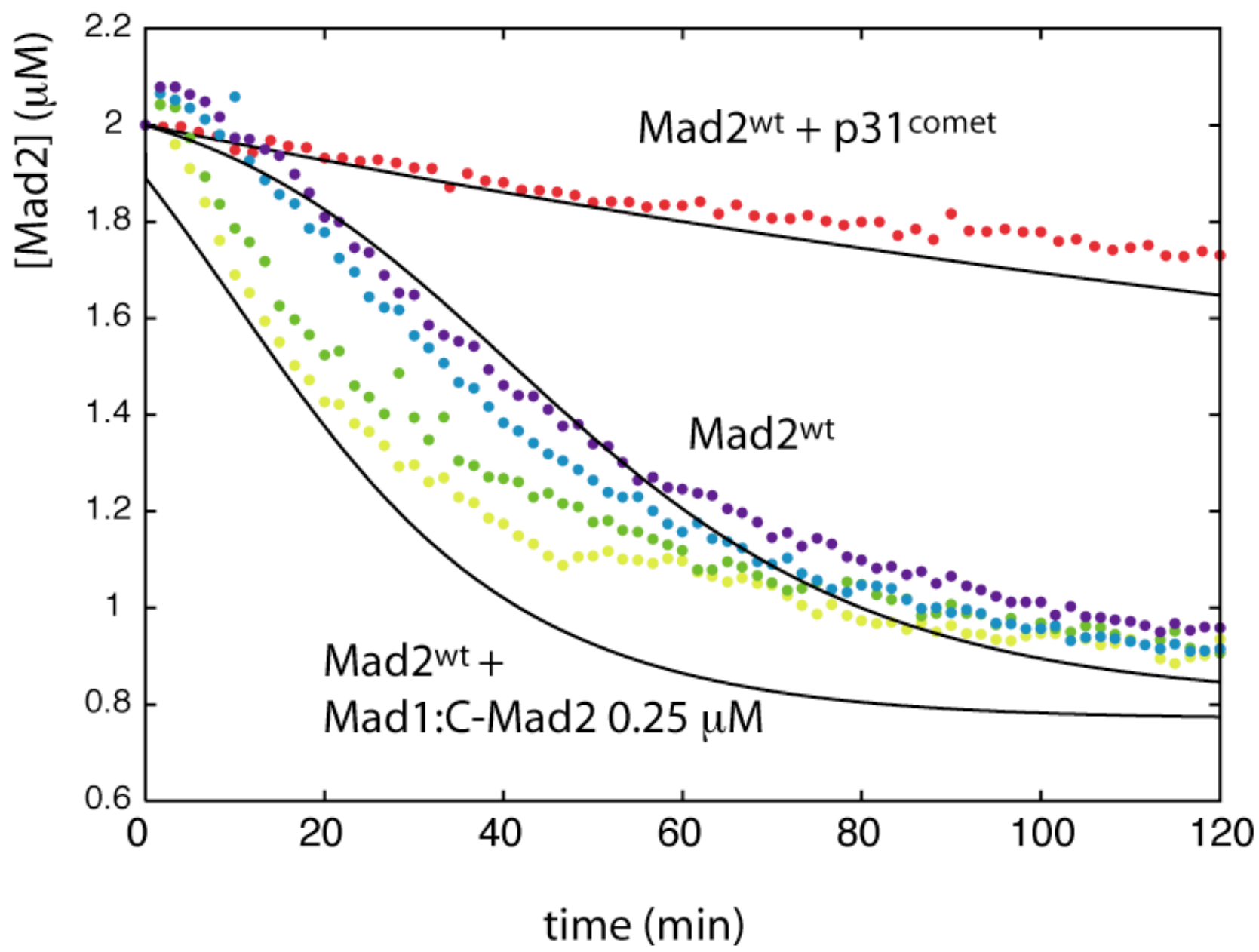

[Mad2] (μM)
2.2
2
1.8
1.6
1.4
1.2
1
0.8
0.6
0
20
40
60
80
100
120
time (min)
Mad2wt + p31comet
Mad2wt
Mad2wt +
Mad1:C-Mad2 0.25 μM
