# Supplementary material for: The Influence of Catalysis on Mad2 Activation Dynamics
Source: PLoS Biol. 2009 Jan 13;7(1):e1000010. doi: 10.1371/journal.pbio.1000010 (PMC2621267; doi:10.1371/journal.pbio.1000010)
Supplement: Supplementary file 5 [file pbio.1000010.sg004.pdf]

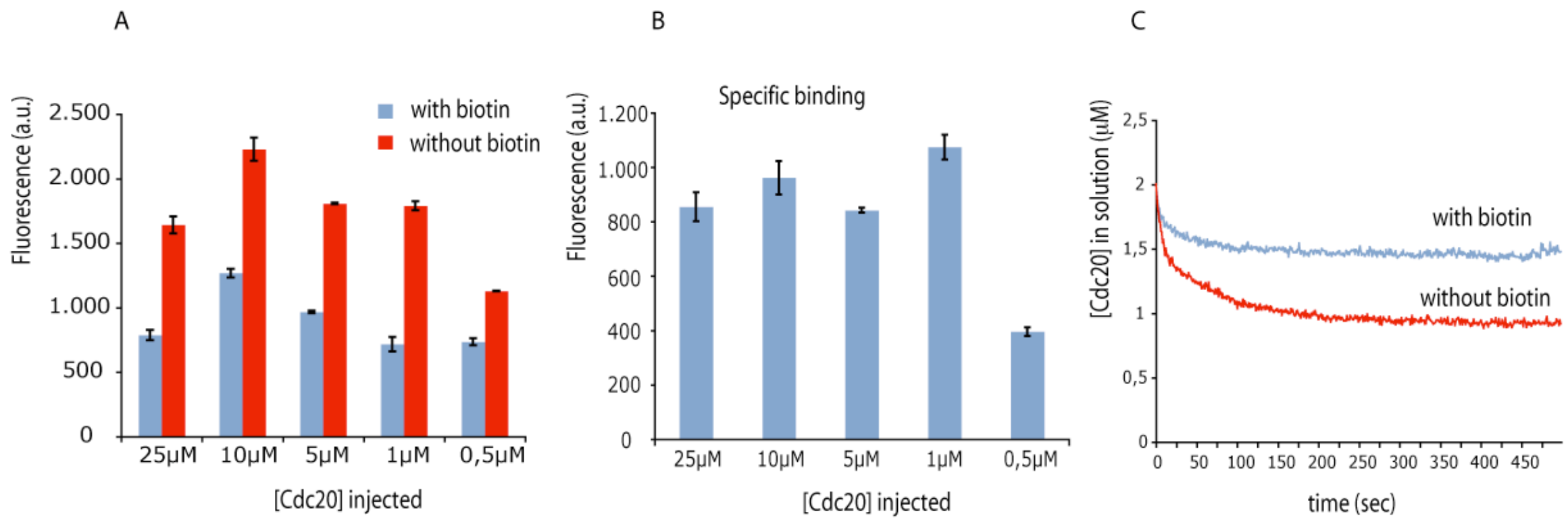

A
B
C
Fluorescence (a.u.)
2.500
2.000
1.500
1.000
500
0
with biotin
without biotin
25μM
10μM
5μM
1μM
0,5μM
[Cdc20] injected
Specific binding
1.200
1.000
800
600
400
200
0
[Cdc20] in solution (μM)
2,5
2
1,5
1
0,5
0
with biotin
without biotin
0
50
100
150
200
250
300
350
400
450
time (sec)
