# Supplementary material for: The Influence of Catalysis on Mad2 Activation Dynamics
Source: PLoS Biol. 2009 Jan 13;7(1):e1000010. doi: 10.1371/journal.pbio.1000010 (PMC2621267; doi:10.1371/journal.pbio.1000010)
Supplement: Supplementary file 6 [file pbio.1000010.sg005.pdf]

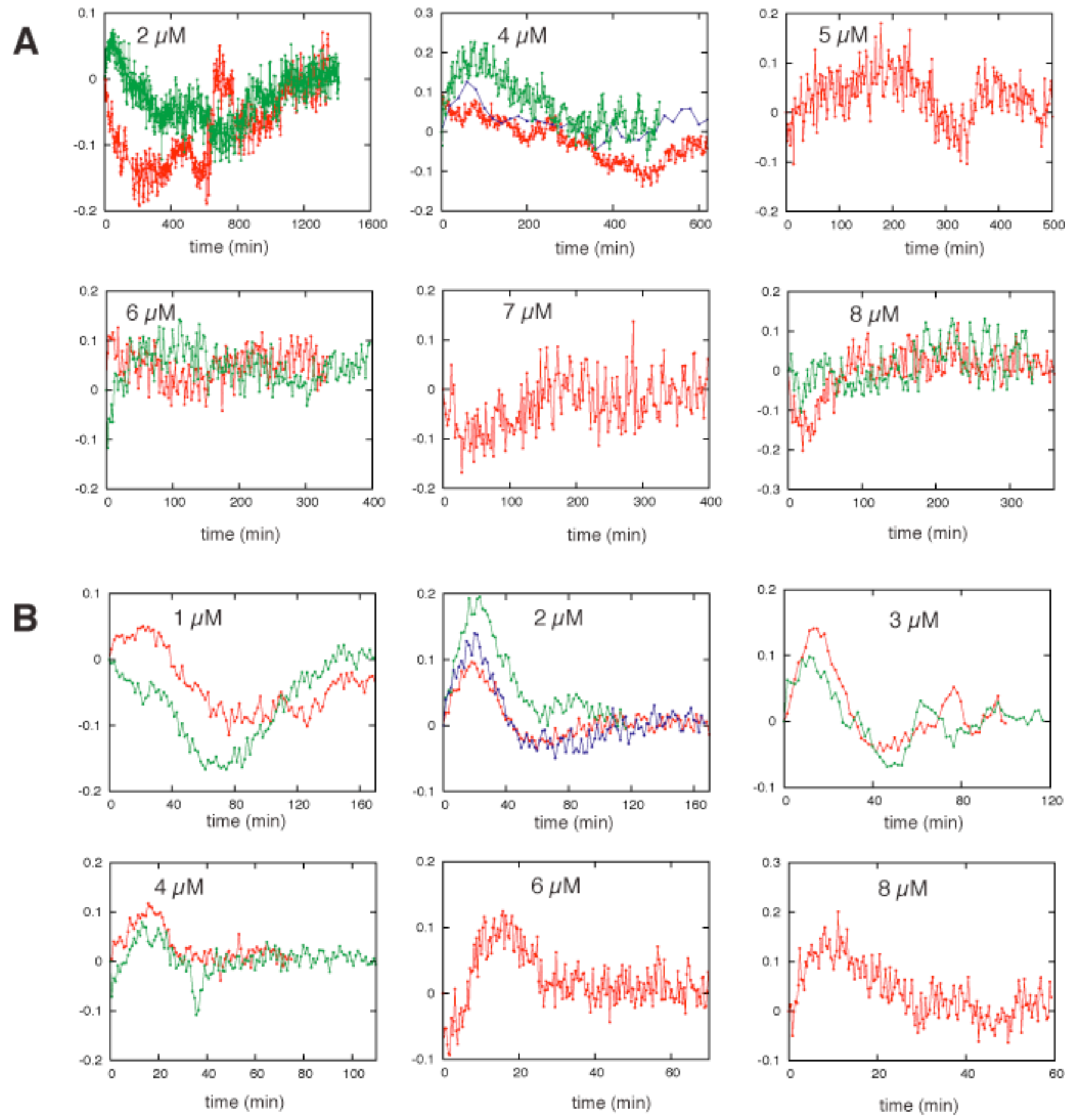
A
2 μM
4 μM
5 μM
6 μM
7 μM
8 μM
B
1 μM
2 μM
3 μM
4 μM
6 μM
8 μM
time (min)
