# Supplementary material for: The Influence of Catalysis on Mad2 Activation Dynamics
Source: PLoS Biol. 2009 Jan 13;7(1):e1000010. doi: 10.1371/journal.pbio.1000010 (PMC2621267; doi:10.1371/journal.pbio.1000010)
Supplement: Supplementary file 7 [file pbio.1000010.st001.pdf]

Algebraic equations for the conservation relations

$$[\text{Mad2}_T]=[\text{Mad2}]+[\text{Cdc20:C-Mad2}]+2\cdot[\text{Cdc20:C-Mad2:O-Mad2}]+[\text{Mad1:C-Mad2:O-Mad2}]+[\text{Cdc20:C-Mad2:p31}^{\text{comet}}]$$

$$[\text{Cdc20}_T]=[\text{Cdc20}]+[\text{Cdc20:C-Mad2}]+[\text{Cdc20:C-Mad2:O-Mad2}]+[\text{Cdc20:C-Mad2:p31}^{\text{comet}}]$$

$$[\text{Mad1:C-Mad2}_T]=[\text{Mad1:C-Mad2}]+[\text{Mad1:C-Mad2:O-Mad2}]$$

$$[\text{p31}_T^{\text{comet}}]=[\text{p31}^{\text{comet}}]+[\text{Cdc20:C-Mad2:p31}^{\text{comet}}]$$

Rates

Reaction 1: $V_{\text{bind}}=k_{\text{bind,on}}[\text{Cdc20}][\text{O-Mad2}]-k_{\text{bind,off}}[\text{Cdc20:C-Mad2}]$

Reaction 2: $V_{\text{m1,dim}}=k_{\text{dim,on}}[\text{Mad1:C-Mad2}][\text{O-Mad2}]-k_{\text{dim,off}}[\text{Mad1:C-Mad2:O-Mad2}]$

Reaction3: $V_{\text{m1,cat}}=k_{\text{cat,on}}[\text{Cdc20}][\text{Mad1:C-Mad1:O-Mad2}]-k_{\text{cat,off}}[\text{Cdc20:C-Mad2}][\text{Mad1:C-Mad2}]$

Reaction 4: $V_{\text{dim}}=k_{\text{dim,on}}[\text{Cdc20:C-Mad2}][\text{O-Mad2}]-k_{\text{dim,off}}[\text{Cdc20:C-Mad2:O-Mad2}]$

Reaction 5: $V_{\text{cat}}=k_{\text{cat,on}}[\text{Cdc20}][\text{Cdc20:C-Mad1:O-Mad2}]-k_{\text{cat,off}}[\text{Cdc20:C-Mad2}]^2$

Reaction 6: $V_{\text{p31}}=k_{\text{bindp31,on}}[\text{Cdc20:C-Mad2}][\text{p31}^{\text{comet}}]-k_{\text{bindp31,off}}[\text{Cdc20:C-Mad2:p31}^{\text{comet}}]$

Differential Equations

1) $$\frac{d[\text{O-Mad2}]}{dt}=-\text{V}_{\text{bind}}-\text{V}_{\text{dim}}-\text{V}_{\text{m1,dim}}$$

2) $$\frac{d[\text{Cdc20}]}{dt}=-\text{V}_{\text{m1,cat}}-\text{V}_{\text{cat}}-\text{V}_{\text{bind}}$$

3) $$\frac{d[\text{Cdc20:C-Mad2}]}{dt}=\text{V}_{\text{bind}}-\text{V}_{\text{dim}}+2\cdot\text{V}_{\text{cat}}+\text{V}_{\text{m1,cat}}-\text{V}_{\text{p31}}$$

4) $$\frac{d[\text{Cdc20:C-Mad2:O-Mad2}]}{dt}=\text{V}_{\text{dim}}-\text{V}_{\text{cat}}$$

5) $$\frac{d[\text{Mad1:C-Mad2:O-Mad2}]}{dt}=\text{V}_{\text{m1,dim}}-\text{V}_{\text{m1,cat}}$$

6) $$\frac{d[\text{Mad1:C-Mad2}]}{dt} = -V_{m1,dim} + V_{m1,cat}$$

7) $$\frac{d[\text{Cdc20:C-Mad2:p31}^{\text{comet}}]}{dt} = V_{p31}$$

8) $$\frac{d[\text{p31}^{\text{comet}}]}{dt} = -V_{p31}$$
