# Supplementary material for: The Influence of Catalysis on Mad2 Activation Dynamics
Source: PLoS Biol. 2009 Jan 13;7(1):e1000010. doi: 10.1371/journal.pbio.1000010 (PMC2621267; doi:10.1371/journal.pbio.1000010)
Supplement: Supplementary file 8 [file pbio.1000010.st002.pdf]

**Chemical Species**	**Reaction 1 (binding)**	**Reaction 2 (dimer formation)**	**Reaction 3 (catalysis)**	**Reaction 4 (dimer formation)**	**Reaction 5 (catalysis)**	**Reaction 6 ($p31^{comet}$ binding)**	**Figure**
$Mad2^{F141A}$	X						3D, 6A S1, S2
$Mad2^{wt}$	X			X	X		4B, 5C, 5D, S1, S2, S3
$Mad2^{wt}$ Mad1:C-Mad2	X	X	X	X	X		5A, 5B, 6A S3
$Mad2^{wt}$ $p31^{comet}$	X			X	X	X	5F, 5G S3
